# Supplementary material for: In Silico Analysis of the Antagonist Effect of Enoxaparin on the ApoE4–Amyloid-Beta (Aβ) Complex at Different pH Conditions
Source: Biomolecules. 2022 Mar 25;12(4):499. doi: 10.3390/biom12040499 (PMC9027285; doi:10.3390/biom12040499)
Supplement: Supplementary file 1 [file biomolecules-12-00499-s001.zip › biomolecules-1558953-supplementary.pdf]

In silico analysis of the competitive antagonist effect of the Enoxaparin on the ApoE4–amyloid-beta (Aβ) complex at different pH conditions

March 24, 2022

Jorge Alberto Aguilar-Pineda[1,3]*, Silvana G. Paco-Coralla[1], Camilo Febres-Molina[2], Pamela L. Gamero-Begazo[3], Pallavi Shrivastava[1], Karin J. Vera-López[1,4], Gonzalo Davila-Del-Carpio[1,5], Patricia López-C[5], Badhin Gómez[3,4], and Christian L. Lino Cardenas[6]*

[1] Laboratory of Genomics and Neurovascular Diseases, Vicerrectorado de Investigación, Universidad Católica de Santa María, Urb. San José s/n—Umacollo, Arequipa 04000, Peru.
[2] Doctorado en Fisicoquímica Molecular, Facultad de Ciencias Exactas, Universidad Andres Bello, Santiago de Chile, 8370134.
[3] Centro de Investigación en Ingeniería Molecular— CIIM, Universidad Católica de Santa María, Urb. San José s/n—Umacollo, Arequipa 04000, Peru.
[4] Facultad de Ciencias Farmacéuticas, Bioquímicas y Biotecnológicas, Universidad Católica de Santa María, Urb. San José s/n—Umacollo, Arequipa 04000, Peru.
[5] Vicerrectorado de Investigación, Universidad Católica de Santa María, Urb. San José s/n—Umacollo, Arequipa 04000, Peru.
[6] Cardiovascular Research Center, Cardiology Division, Massachusetts General Hospital, Boston, MA 02114, USA

Keywords: Alzheimer disease, Apolipoprotein E, amyloid-β, Enoxaparin, Molecular Dynamics.

*Author to whom correspondence should be addressed.
Electronic mail: Clinocardenas@mgh.harvard.edu & jaguilar@ucsm.edu.pe

Supplementary Figures

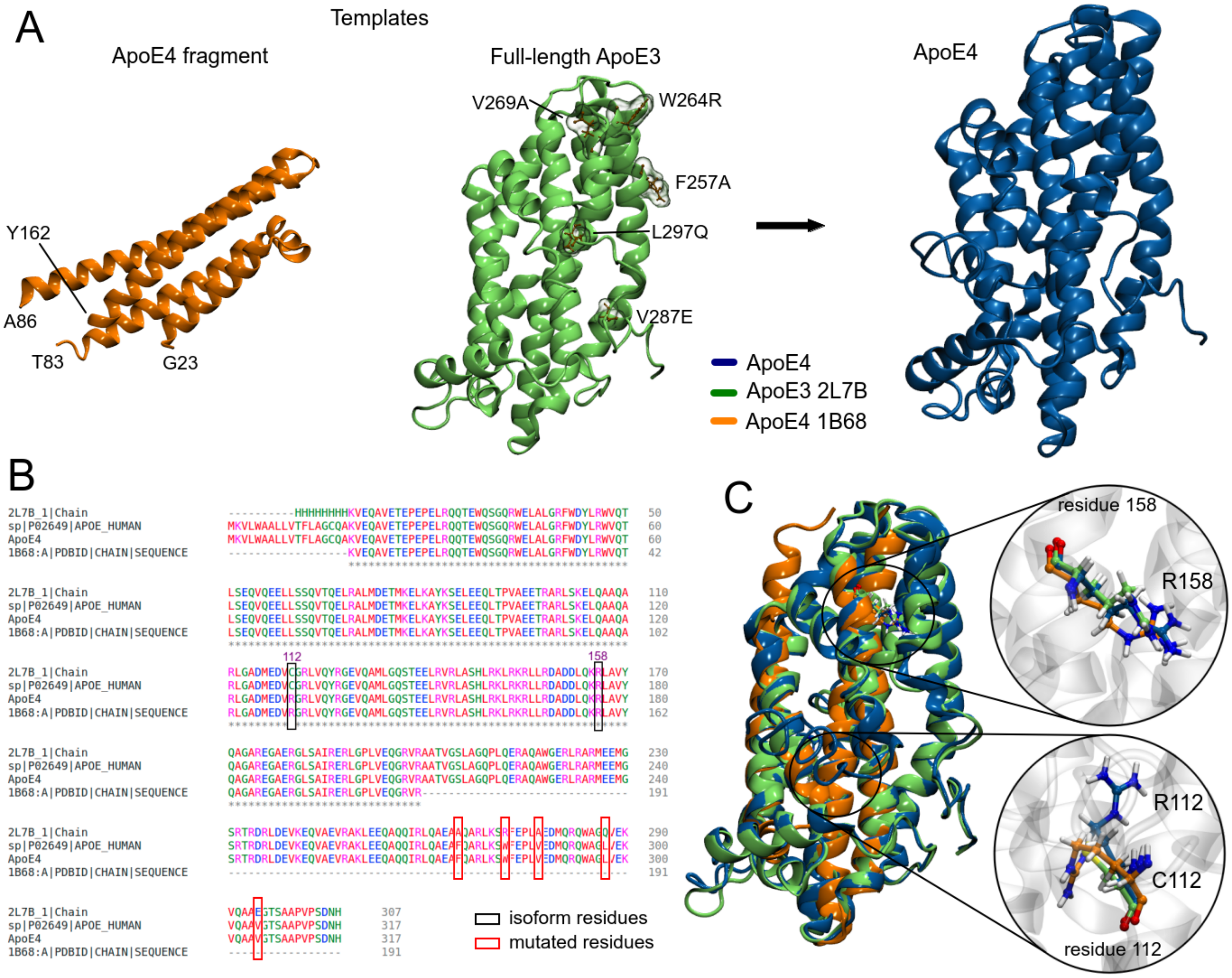

Figure S1. Obtaining the structure of the ApoE4 protein through modeling techniques. (A) Structure models of a fragment of ApoE4 (PDB ID: 1B68) and mutated ApoE3 (PDB ID: 2L7B) were chosen as templates. (B) FASTA sequence alignment, where mutated residues are indicated. (C) Alignment of the ApoE4 structure with the templates, the difference between the ApoE4 structure and the ApoE3 structure is at amino acid position 112 (arginine and cysteine).

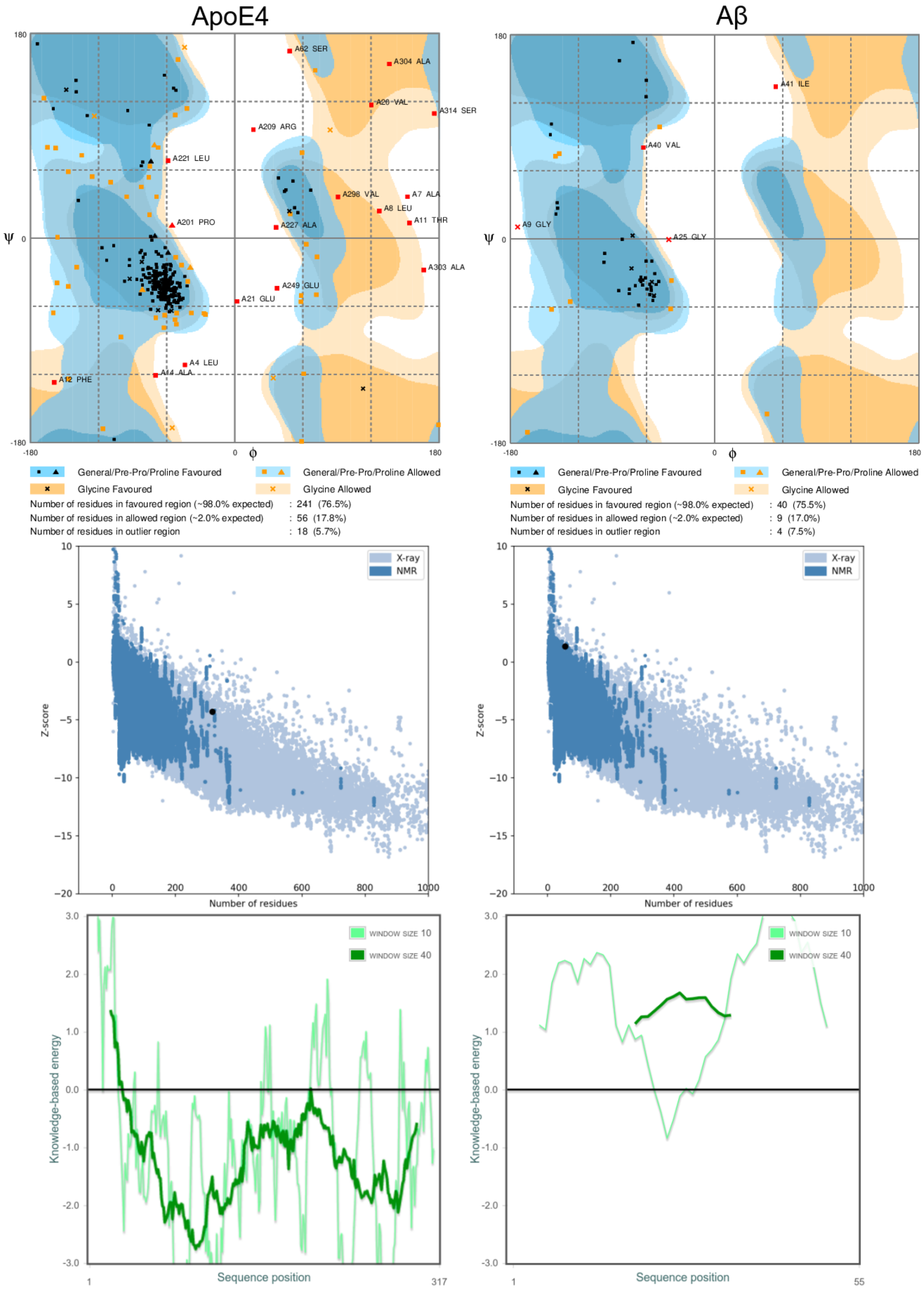

Figure S2. PDBSum Results of the ApoE4 and Aβ structures.

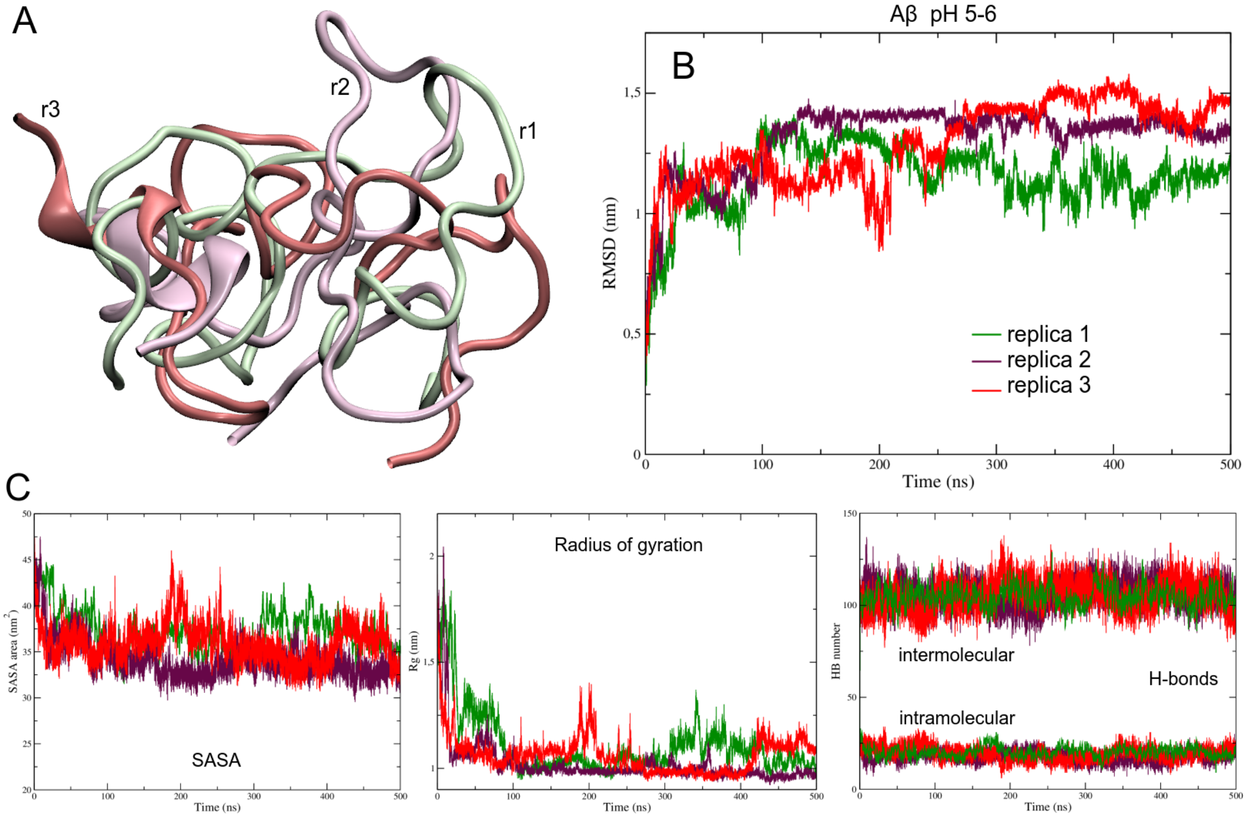

Figure S3. Final Aβ structures obtained in MD simulations with 500 ns trajectories under pH5 - 6 conditions. The structure used in this work to obtain the ApoE4-Aβ complexes is shown in green. The structures in red and purple are replicas of the Aβ peptide, both structures tend to have a globular shape.

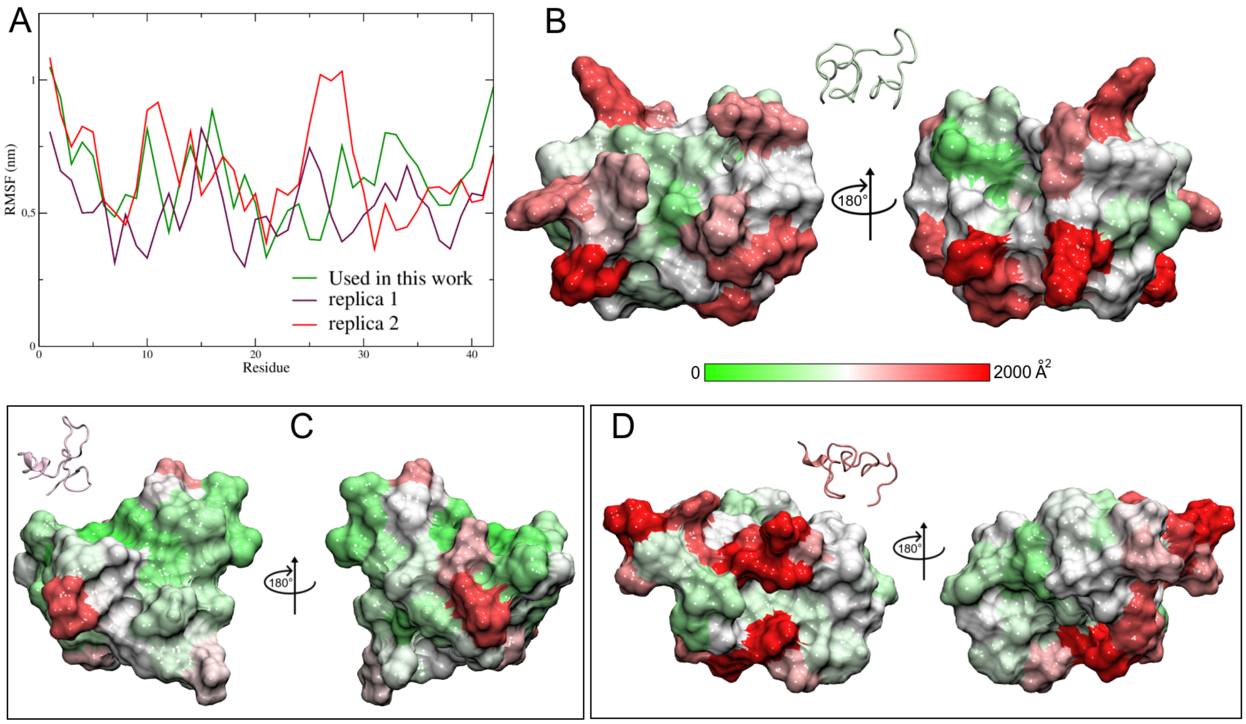

Figure S4. Vibrational analysis of Aβ structures at pH5 - 6. (A) The RMSF plot shows the fluctuation of the residues with respect to their equilibrium position. High values in its standard deviation are characteristic of intrinsically disordered structures. At this pH, the structures lose α-helices. (B, C, and D) Molecular surfaces representing factor B, values in red indicate high molecular vibration; white, medium vibrations; while green shows low vibrations. Structures B (used in this work) and D, showed a higher vibration of their residues.

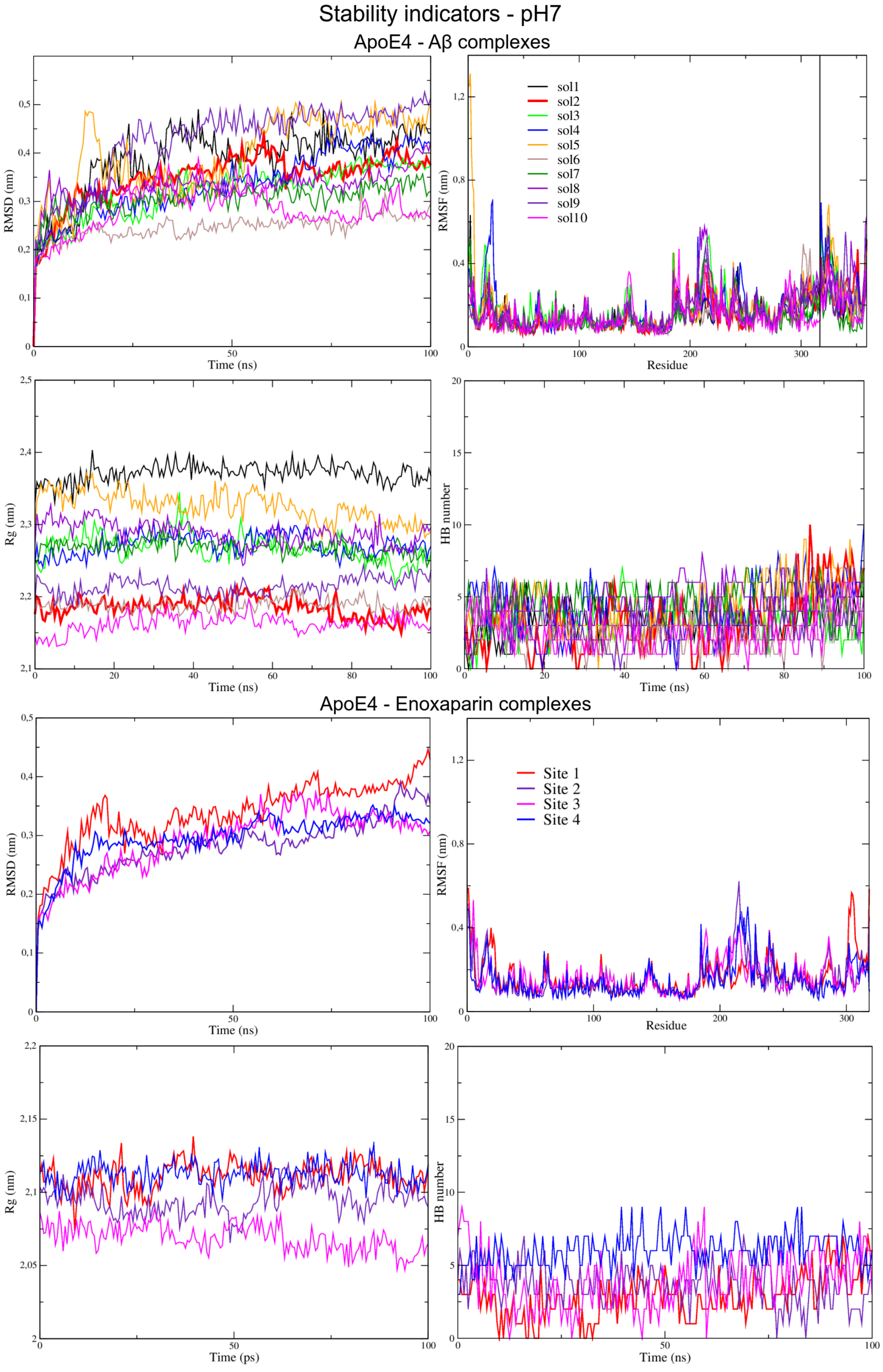

Figure S5. Structural stability indicators for the ApoE4 ligand systems at pH7 were analyzed in this work. For the interactions with Aβ, the 10 best solutions obtained by molecular docking using the FireDock server were chosen, which performs a flexible refinement according to an energy function. For interactions with Enx, local docking was performed at the different binding sites found on the surface of ApoE4 using AutoDock Vina software. The highlighted solution (red line) corresponds to the structure with the highest energy affinity.

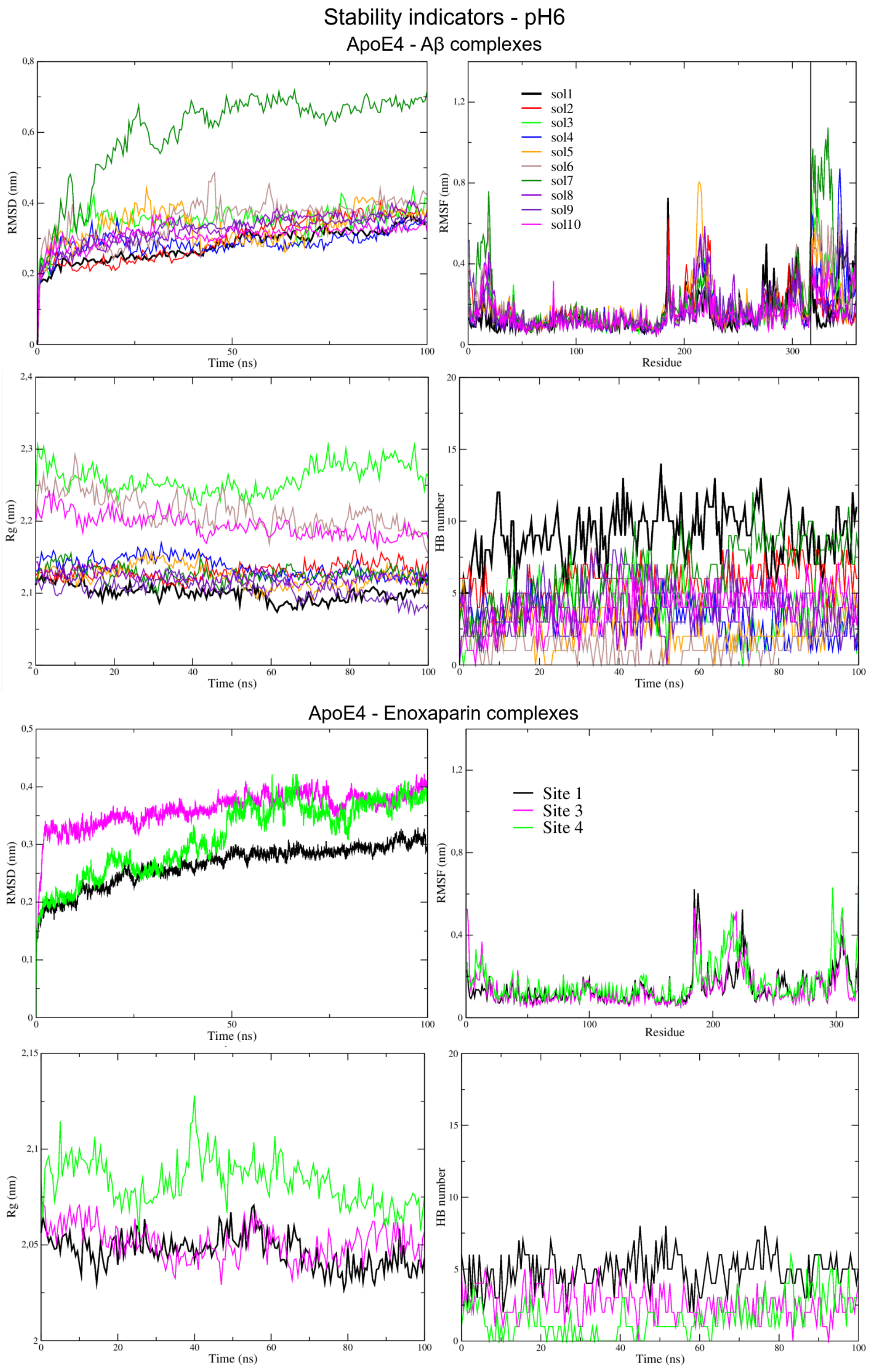

Figure S6. Structural stability indicators for the ApoE4 ligand systems at pH6 were analyzed in this work. For the interactions with Aβ, the 10 best solutions obtained by molecular docking using the FireDock server were chosen, which performs a flexible refinement according to an energy function. For interactions with Enx, local docking was performed at the different binding sites found on the surface of ApoE4 using AutoDock Vina software. The highlighted solution (black line) corresponds to the structure with the highest energy affinity.

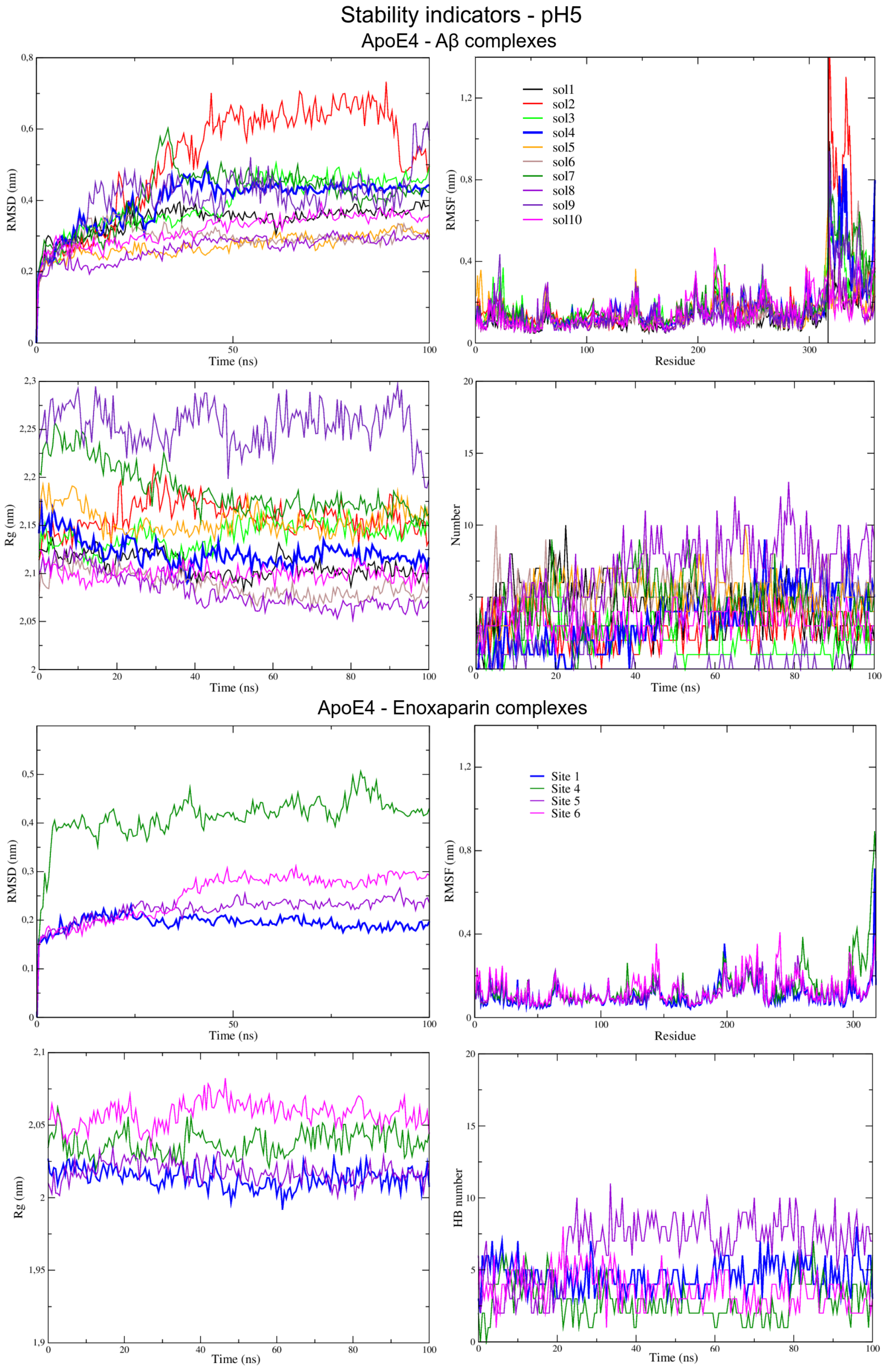

Figure S7. Structural stability indicators for the ApoE4 ligand systems at pH5 were analyzed in this work. For the interactions with Aβ, the 10 best solutions obtained by molecular docking using the FireDock server were chosen, which performs a flexible refinement according to an energy function. For interactions with Enx, local docking was performed at the different binding sites found on the surface of ApoE4 using AutoDock Vina software. The highlighted solution (blue line) corresponds to the structure with the highest energy affinity.

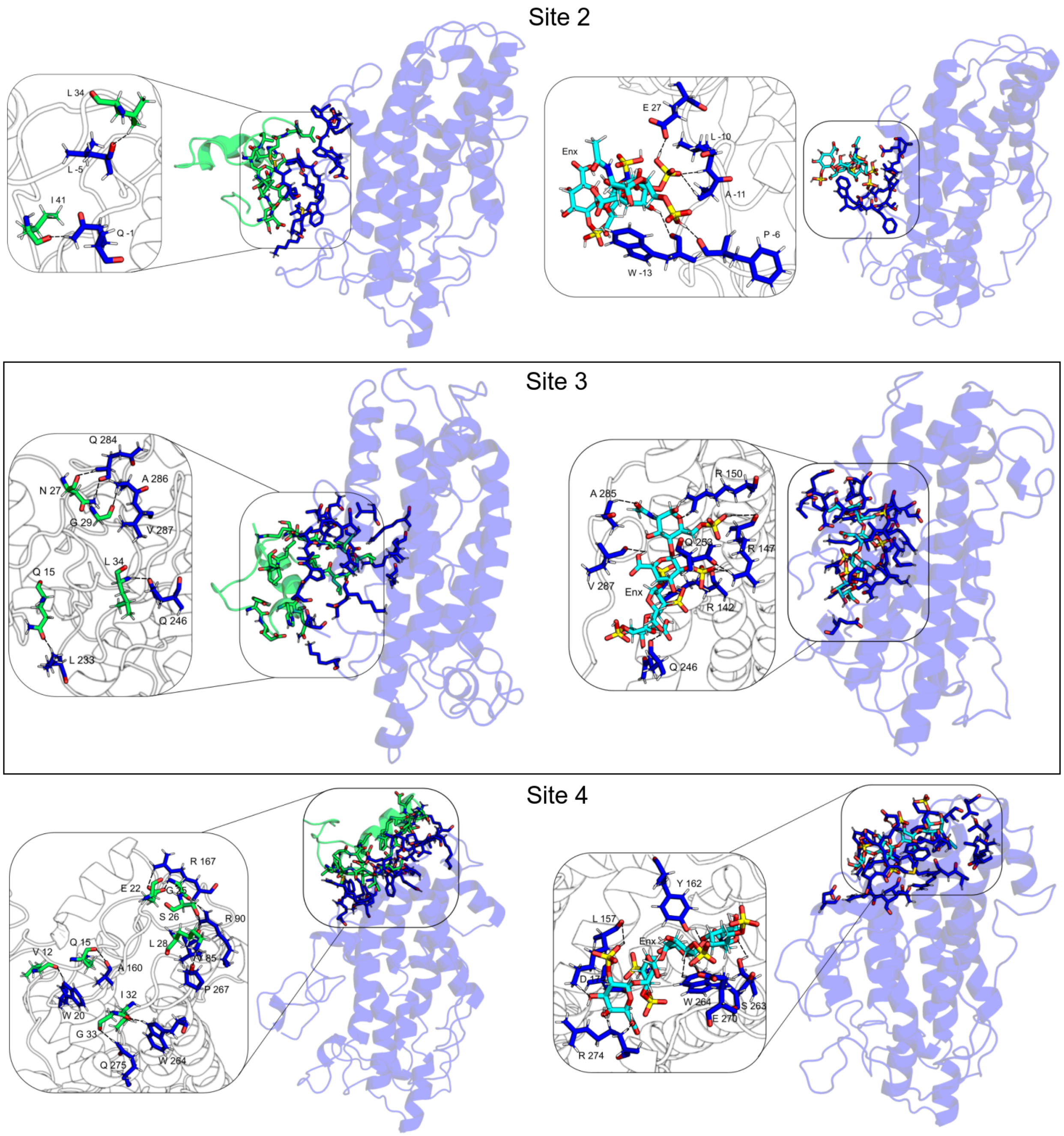

Figure S8. Structures of molecular protein-ligand interaction complexes at pH7 binding sites. For each site, complexes with the highest protein-ligand affinity were chosen. The ApoE4-Aβ interaction is shown in the left panels and the ApoE4-Enx interaction in the right panels. ApoE4 residues are shown in blue, while for ligands, green was used for Aβ residues and cyan for Enx.

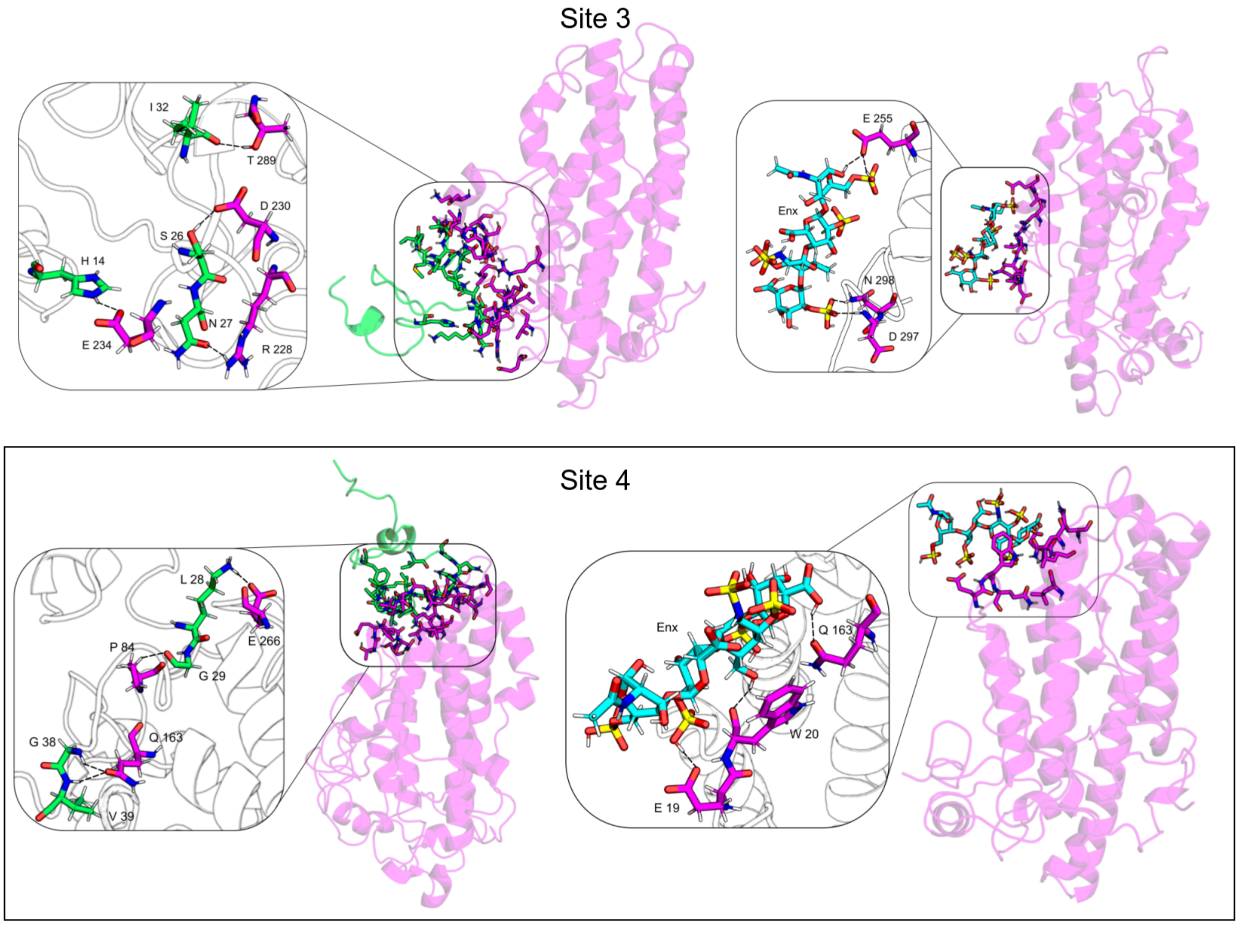

Figure S9. Structures of molecular protein-ligand interaction complexes at pH6 binding sites. For each site, complexes with the highest protein-ligand affinity were chosen. The ApoE4-Aβ interaction is shown in the left panels and the ApoE4-Enx interaction in the right panels. ApoE4 residues are shown in magenta, while for ligands, green was used for Aβ residues and cyan for Enx.

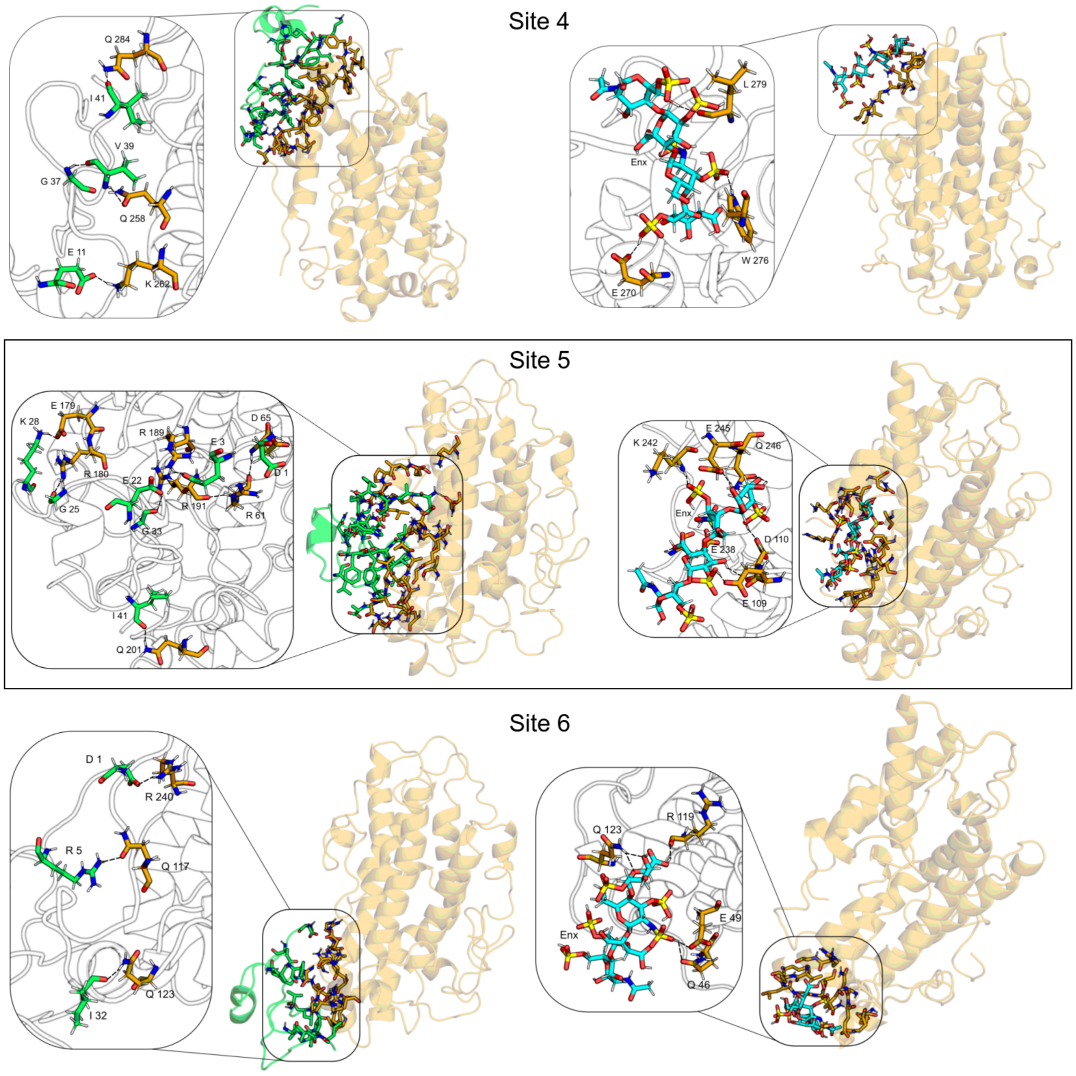

Figure S10. Structures of molecular protein-ligand interaction complexes at pH5 binding sites. For each site, complexes with the highest protein-ligand affinity were chosen. The ApoE4-Aβ interaction is shown in the left panels and the ApoE4-Enx interaction in the right panels. ApoE4 residues are shown in orange, while for ligands, green was used for Aβ residues and cyan for Enx.

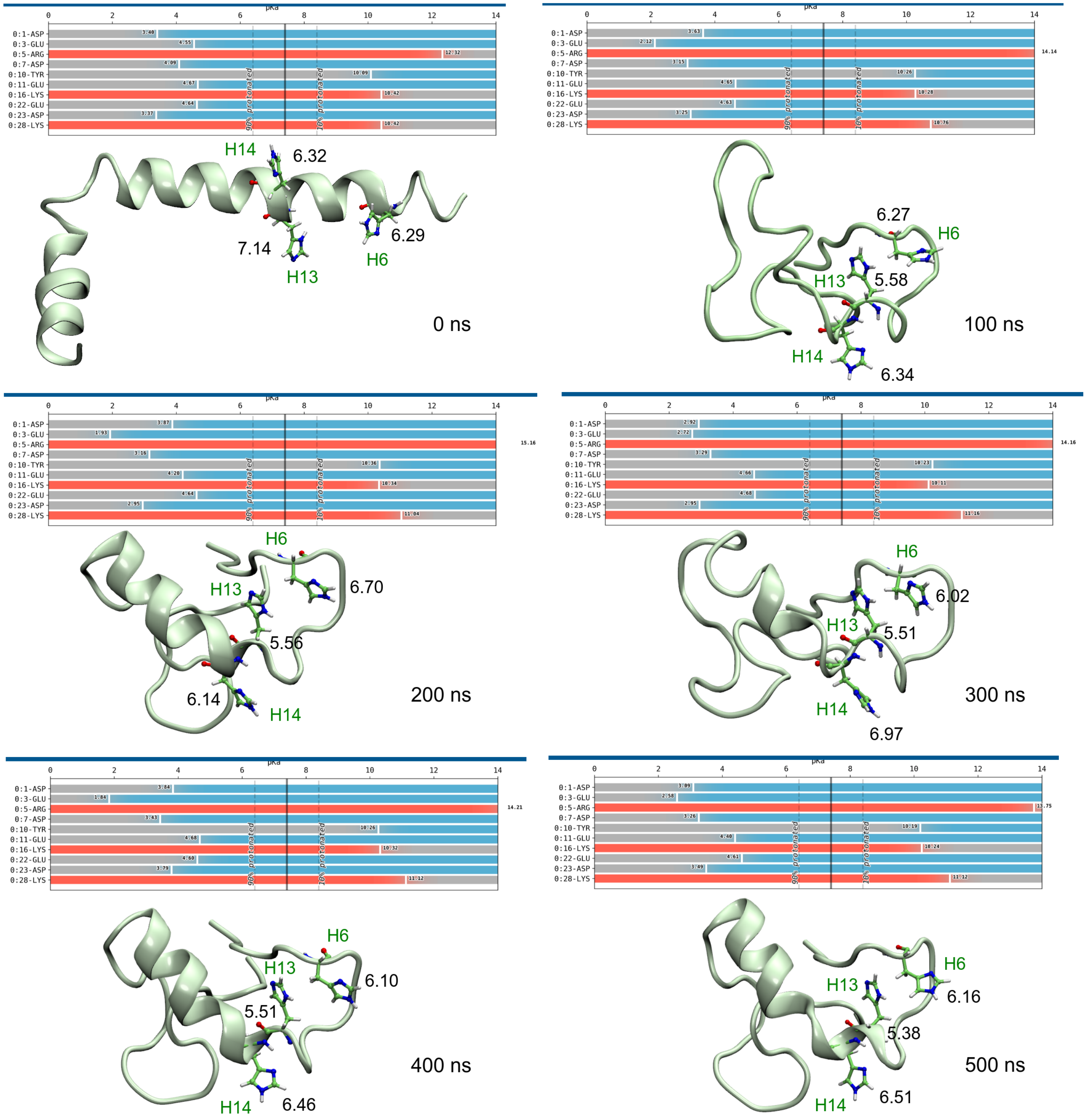

Figure S11. Analysis of pKa values obtained in PROPKA calculations at every 100 ns of the MD simulation of Aβ at neutral pH. The tables show the pKa values of the ionizable residues Asp, Glu, Tyr, Lys, and Arg, while the 3D figures show the pKa values of His6, His13, and His14 for each case.

Table S1. pKa values of the ApoE4 and Aβ ionizable residues obtained in PROPKA calculations.

Residue	ApoE4	Aβ
Asp	**35(6.82)**[a], **65(5.04)**, 107(4.66), **110(5.50)**, **151(6.40)**, **153(8.03)**, 154(2.57), **227(5.12)**, 230(4.02), 271(4.62), 297(4.35)	1(3.98), 7(4.07), 23(3.37)
Glu	3(4.62), **7(5.33)**, **9(5.58)**, **11(5.53)**, 13(4.73), 19(4.84), **27(6.92)**, 45(4.55), 49(4.82), **50(6.33)**, **59(7.25)**, 66(4.46), 70(4.68), 77(4.58), **79(7.76)**, 80(4.76), 87(4.90), 88(4.38), **96(5.72)**, 109(4.26), 121(4.97), 131(4.36), **132(5.85)**, 168(4.25), 171(4.62), 179(4.72), **186(5.35)**, 205(4.61), 212(4.35), 219(4.50), 220(3.76), 231(4.52), 234(4.22), 238(3.50), 244(4.51), **245(6.85)**, 255(4.36), 266(3.97), 270(4.69), 281(4.74)	3(4.55), 11(4.67), 22(4.64)
His	**140(5.05)**, **299(6.33)**	**6(6.29), 13(7.14), 14(6.32)**
Cys	-2(9.53)	-
Tyr	36(11.00), 74(10.00), 118(12.95), 162(15.00)	10(10.11)
Lys	-16(10.45), 1(8.30), 69(10.64), 72(10.50), 75(9.76), 95(10.33), 143(9.56), 146(9.13), 157(10.01), 233(10.00), 242(12.28), 262(11.30), 282(10.34)	16(10.42), 28(10.42)
Arg	15(12.77), 25(13.62), 32(12.82), 38(9.27), 61(11.83), 90(10.81), 92(12.61), 103(11.37), 112(11.91), 114(10.45), 119(11.31), 134(10.43), 136(10.83), 142(9.71), 145(12.12), 147(10.57), 150(8.69), 158(12.28), 167(12.45), 172(12.48), 178(12.36), 180(11.95), 189(12.37), 191(13.01), 206(12.06), 213(12.96), 215(12.69), 217(11.54), 224(13.27), 226(12.08), 228(12.37), (240(12.54), 251(12.62), 260(13.54), 274(12.21)	5(12.36)

[a] $b(c)$, b=protein sequence number, c=pKa value. The protonated residues used in this work are bold.

Table S2. Quality of the models of ApoE4 structures, using the I-TASSER server.

**ApoE4 models**					
No. Model	C-Score	TM-Score	RMSD	No. of decoys	Cluster density
1	-0.16	0.69±0.12	6.7±4.0Å	7001	0.2671
2	-0.66	-	-	4094	0.1625
3	-2.39	-	-	768	0.0288
4	-0.23	-	-	4750	0.2504
5	-3.30	-	-	94	0.0115

Table S3. Stability indicators for ApoE4-Aβ complexes at pH7.

**ApoE4-Aβ Interaction pH7**				
System	RMSD	RMSF	RG	HB
Sol1	0.38±0.07	0.17±0.08	2.37±0.01	3.16±1.11
Sol2	0.35±0.05	0.16±0.09	2.19±0.01	3.36±1.96
Sol3	0.32±0.05	0.19±0.10	2.27±0.02	3.29±1.17
Sol4	0.34±0.06	0.18±0.11	2.27±0.01	4.03±1.79
Sol5	0.40±0.08	0.19±0.14	2.33±0.02	4.36±1.71
Sol6	0.25±0.02	0.15±0.08	2.19±0.01	2.31±1.37
Sol7	0.31±0.03	0.15±0.08	2.27±0.01	4.87±1.05
Sol8	0.33±0.03	0.18±0.10	2.29±0.02	4.60±1.23
Sol9	0.43±0.08	0.18±0.09	2.21±0.01	2.85±1.19
Sol10	0.28±0.04	0.16±0.08	2.16±0.01	2.43±1.25

Table S4. Stability indicators for ApoE4-Enx complexes at pH7.

**ApoE4-Enx interactions pH7**				
System	RMSD	RMSF	RG	HB
Site 1	0.35±0.06	0.17±0.09	2.11±0.01	2.95±1.41
Site 2	0.28±0.05	0.15±0.09	2.09±0.01	3.61±1.36
Site 3	0.29±0.05	0.17±0.08	2.07±0.01	3.71±1.92
Site 4	0.30±0.04	0.15±0.08	2.11±0.01	6.14±1.18

Table S5. Stability indicators for ApoE4-Aβ complexes at pH6.

**ApoE4-Aβ Interaction pH6**				
System	RMSD	RMSF	RG	HB
Sol1	0.28±0.04	0.16±0.10	2.10±0.01	8.97±1.90
Sol2	0.30±0.06	0.17±0.10	2.13±0.01	5.68±1.37
Sol3	0.35±0.04	0.17±0.11	2.26±0.02	3.79±1.70
Sol5	0.33±0.05	0.18±0.12	2.12±0.01	2.44±1.22
Sol6	0.35±0.05	0.21±0.14	2.21±0.02	1.28±1.01
Sol7	0.60±0.12	0.22±0.18	2.13±0.01	6.28±2.34
Sol8	0.33±0.04	0.16±0.09	2.12±0.01	3.68±1.15
Sol9	0.32±0.04	0.17±0.09	2.11±0.01	4.34±1.92
Sol10	0.31±0.03	0.15±0.07	2.19±0.02	4.11±1.55

Table S6. Stability indicators for ApoE4-Enx complexes at pH6.

**ApoE4-Enx interactions pH6**				
System	RMSD	RMSF	RG	HB
Site 1	0.26±0.04	0.14±0.08	2.05±0.01	4.84±1.09
Site 3	0.36±0.03	0.14±0.09	2.05±0.01	2.55±1.09
Site 4	0.31±0.06	0.17±0.10	2.08±0.01	1.55±1.30

Table S7. Stability indicators for ApoE4-Aβ complexes at pH5.

**ApoE4-Aβ Interaction pH5**				
System	RMSD	RMSF	RG	HB
Sol1	0.35±0.04	0.13±0.09	2.11±0.01	4.28±1.82
Sol2	0.52±0.15	0.21±0.20	2.16±0.02	2.81±0.20
Sol3	0.39±0.07	0.18±0.11	2.14±0.01	1.80±1.00
Sol4	0.40±0.07	0.18±0.13	2.12±0.01	3.43±2.04
Sol5	0.27±0.03	0.15±0.08	2.15±0.01	5.00±1.43
Sol6	0.30±0.03	0.14±0.09	2.09±0.01	5.23±1.33
Sol7	0.41±0.08	0.19±0.13	2.19±0.03	4.26±1.79
Sol8	0.27±0.03	0.14±0.07	2.08±0.02	7.04±2.35
Sol9	0.41±0.07	0.17±0.12	2.25±0.02	0.59±0.89
Sol10	0.32±0.04	0.15±0.08	2.10±0.01	3.28±1.11

Table S8. Stability indicators for ApoE4-Enx complexes at pH5.

**ApoE4-Enx interactions pH5**				
System	RMSD	RMSF	RG	HB
Site 1	0.19±0.01	0.11±0.06	2.01±0.01	4.52±1.11
Site 4	0.41±0.04	0.14±0.10	2.04±0.01	2.83±1.24
Site 5	0.22±0.02	0.12±0.05	2.02±0.01	6.70±2.27
Site 6	0.25±0.04	0.13±0.07	2.06±0.01	3.59±1.23

Table S9. Residues involved in the ApoE4-Aβ interactions at pH7. The molecular systems were analyzed after 100 ns of MD simulations.

Site	ApoE4		Aβ	
	**H-bonds**	**Hydrophobic contacts**	**H-bonds**	**Hydrophobic contacts**
1	M-17, R25, R32, D35, L214	A-12, W-13, V-15, Q24, L28, G211, E212, R213, R215	E11, Q15, D23, G33	G9, V12, K16, V18, F19, E22, I31, I32, L34
2	L-5, E-1	M-17, V-15 W-13, A-12, P10, E11, P12	L34, I41	Y10, A30, G33, M35, V39, A42
3	K233, Q246, Q284, A286, V287	K143, R147, R150, I250, L279, E281, V283, A285, P293, V294, P295, S296, N298	Q15, N27, G29, L34	H14, A30, I31, I32, G33, M35, V39, I41
4	W20, V85, R90, A160, Q163, R167, W264, P267, Q275	T83, P84, L157, V162, A164, G165, S263, L268	V12, Q15, L16, E22, G25, S26, K28, I32, G33	H14, V18, F19, A21, D23, D27, G29, A30, I31, V36

Table S10. ApoE4 residues involved in the ApoE4-Enx interactions at pH7. The molecular systems were analyzed after 100 ns of MD simulations.

Site	ApoE4	
	**H-bonds**	**Hydrophobic contacts**
1	M-17, A-12, L-10, D35, R215	K-16, V-15, L-14, A-11, L28, R32
2	W-13, A-11, L-10, F-6, E27	A-12, V-8
3	R142, R147, R150, Q246, Q253, A285, V287	K143, Q231, Q249, I250, L279, E281, V283, Q284, G288, P295
4	D154, K157, Y162, S263, W264, E270, R274	T89, L93, R158, V161, L268, D271, M272, Q273, Q275

Table S11. Residues involved in the ApoE4-Aβ interactions at pH6. The molecular systems were analyzed after 100 ns of MD simulations.

**Site**	**ApoE4**		**Aβ**	
	**H-bonds**	**Hydrophobic contacts**	**H-bonds**	**Hydrophobic contacts**
1	Q24, D35, R206, G211, R215, R217	V-15, L-14, W-13, L28, G31, W39, E50, E205, Q208, A209, W210, L214, M218	D7, S8, Y10, H14, K16, D23	F4, R5, G9, E11, H13, Q15, V18, G25, S26, V39, I41, A42
3	R288, D230, E234, T289	D227, E231, V232, K282, A285, A286, V287, G288, S290, H299	H14, S26, N27, I32	G29, A30, G33, V36, V40, I41, A42
4	P84, Q163, E266	T18, E19, W20, E77, Q81, T83, V85, A86, T89, A160, A164, P267, L268	K28, G29, G38, V39	F20, V24, S26, N27, A30, I32, G33, V36, G37, V40

Table S12. ApoE4 residues involved in the ApoE4-Enx interactions at pH6. The molecular systems were analyzed after 100 ns of MD simulations.

**Site**	**ApoE4**	
	**H-bonds**	**Hydrophobic contacts**
1	R15, E27, D35, E50, E205	L-14, W-13, L-10, L28, G31, W39, W210, L214
3	E255, D297, N298	R251, A254, V294, P295, S296
4	E19, W20, Q163	E77, L78, Q81

Table S13. Residues involved in the ApoE4-Aβ interactions at pH5. The molecular systems were analyzed after 100 ns of MD simulations.

Site	ApoE4		Aβ	
	**H-bonds**	**Hydrophobic contacts**	**H-bonds**	**Hydrophobic contacts**
1	K-16, L-14, L-10, R15, E19	M-17, V-15, A-11, L-5, A-4, P12, Q16, W20, L28	R5, I32, G33,V40, A42	K16, L17, V18 ,F19, G29, A30, L34, M35, V36, V39, I41
4	Q258, K262, Q284	F257, L261, W264, F265, W276, A277, G278, L279, V287, G28, T289, S290, A291	E11, G37, V39, I41	H14, Q15, K16, L17, F20, K28, G29, A30, I31, G33, G38, V40, A42
5	R61, D65, E179, R180, R189, R191, Q201	T57, E109, R112, G113, V116, L181, P183, V185, E186, A192, T194, L198, G200	D1, E3, E22, G25, K28, G33, I41	A2, F4, H6, F19, F20, V24, I31, I32, L34, V36, G37, V40
6	Q117, Q123, R240	G120, Q201, P202, L203, A237	D1, R5, I32	I31, G33, L34, V36, V39, I41

Table S14. ApoE4 residues involved in the ApoE4-Enx interactions at pH5. The molecular systems were analyzed after 100 ns of MD simulations.

Site	ApoE4	
	**H-bonds**	**Hydrophobic contacts**
1	A-12, L-10, T18, E19, Q24	M-17, K-16, V-15, L-14, A-11, L-5
4	E270, W276, L279	V269, Q273, G278
5	E109, D110, E238, K242, E245, Q246	A106, G113, Q117, R180, A241, Q249
6	Q46, E49, R119, Q123	E45, Q48, L52, G120, V122, L126, G127, Q128

Table S15. Top 10 residues that contribute to the binding free energy in the ApoE4 and Aβ protein structures at pH7.

No.	Site S2			Site S3			Site S4		
	**ApoE4$_{A\beta}$**	**Aβ**	**ApoE4$_{Enx}$**	**ApoE4$_{A\beta}$**	**Aβ**	**ApoE4$_{Enx}$**	**ApoE4$_{A\beta}$**	**Aβ**	**ApoE4$_{Enx}$**
1	M-17(**-294**)	L34(**-107**)	E27(**-30**)	K233(**-123**)	I32(**-138**)	K143(**-21**)	R167(**-256**)	I32(**-137**)	K157(**-87**)
2	K-16(**-222**)	I41(**-71**)	Q24(**-24**)	K282(**-113**)	I31(**-115**)	R147(**-11**)	P84(**-163**)	K16(**-125**)	R274(**-47**)
3	L-5(**-129**)	I32(**-70**)	R15(**-23**)	K146(**-107**)	L34(**-98**)	R142(**-9**)	R90(**-155**)	I31(**-95**)	W264(**-45**)
4	R213(**-119**)	H14(**-64**)	P12(**-21**)	K1(**-106**)	Q15(**-84**)	Q253(**-7**)	W20(**-151**)	V12(**-87**)	D154(**-40**)
5	K1(**-108**)	Y10(**-59**)	E19(**-17**)	R228(**-105**)	V18(**-64**)	S22(**-5**)	K157(**-131**)	L34(**-69**)	V161(**-32**)
6	R15(**-104**)	S8(**-57**)	T18(**-14**)	K143(**-100**)	H14(**-59**)	Q24(**-5**)	R274(**-96**)	Q15(**-63**)	Y162(**-28**)
7	R226(**-102**)	V39(**-48**)	L28(**-12**)	R240(**-99**)	M35(**-56**)	R213(**-5**)	V161(**-95**)	V36(**-62**)	158(**-21**)
8	R215(**-96**)	A42(**-47**)	L-10(**-10**)	R251(**-94**)	N27(**-45**)	K282(**-5**)	A164(**-91**)	A30(**-56**)	E270(**-17**)
9	C-2(**-96**)	M35(**-38**)	R206(**-6**)	R142(**-91**)	G33(**-45**)	R145(**-4**)	R172(**-83**)	V18(**-55**)	R260(**-16**)
10	R228(**-95**)	G33(**-32**)	Q46(**-6**)	R147(**-86**)	A30(**-32**)	K233(**-4**)	R158(**-71**)	G33(**-52**)	S263(**-13**)

Values in bold and parentheses are BFE per residue in kJ·mol^{-1}. Aβ and Enx suffix indicates the molecular complex

Table S16. Top 10 residues that contribute to the binding free energy in the ApoE4 and Aβ protein structures at pH6.

No.	Site S3			Site S4		
	**ApoE4$_{A\beta}$**	**Aβ**	**ApoE4$_{Enx}$**	**ApoE4$_{A\beta}$**	**Aβ**	**ApoE4$_{Enx}$**
1	H299(**-165**)	H14(**-162**)	P295(**-74**)	R167(**-162**)	K28(**-120**)	W20(**-160**)
2	E234(**-111**)	I32(**-129**)	V294(**-73**)	E266(**-134**)	H14(**-115**)	E19(**-101**)
3	A286(**-78**)	A42(**-91**)	N298(**-72**)	W20(**-116**)	I32(**-112**)	Q163(**-34**)
4	R228(**-75**)	I41(**-91**)	R251(**-60**)	P267(**-111**)	I41(**-102**)	Q17(**-17**)
5	V232(**-72**)	S26(**-86**)	S296(**-53**)	E19(**-90**)	H6(**-101**)	Q81(**-14**)
6	D230(**-69**)	G29(**-58**)	E255(**-38**)	A164(**-85**)	R5(**-93**)	E13(**-13**)
7	K282(**-68**)	A30(**-57**)	A254(**-26**)	L268(**-83**)	A30(**-73**)	E77(**-13**)
8	V2(**-53**)	G33(**-54**)	H299(**-18**)	P84(**-68**)	G33(**-53**)	L78(**-12**)
9	T289(**-49**)	Q15(**-45**)	L243(**-7**)	Q81(**-55**)	V36(**-51**)	A160(**-9**)
10	A291(**-40**)	R5(**-37**)	R240(**-6**)	Q163(**-47**)	G29(**-50**)	Q21(**-6**)

Values in bold and parentheses are BFE per residue in kJ·mol^{-1}. Aβ and Enx suffix indicates the molecular complex

Table S17. Top 10 residues that contribute to the binding free energy in the ApoE4 and Aβ protein structures at pH5.

No.	Site S4			Site S5			Site S6		
	**ApoE4$_{A\beta}$**	**Aβ**	**ApoE4$_{Enx}$**	**ApoE4$_{A\beta}$**	**Aβ**	**ApoE4$_{Enx}$**	**ApoE4$_{A\beta}$**	**Aβ**	**ApoE4$_{Enx}$**
1	K262(**-274**)	E11(**-122**)	V6(**-101**)	R189(**-266**)	E22(**-234**)	D110(**-69**)	P202(**-137**)	E3(**-135**)	E45(**-125**)
2	L261(**-143**)	I41(**-122**)	E7(**-64**)	R61(**-158**)	E3(**-213**)	E109(**-53**)	Q123(**-131**)	F20(**-121**)	Q48(**-104**)
3	F265(**-126**)	A42(**-78**)	Q4(**-57**)	R191(**-137**)	D23(**-143**)	E238(**-40**)	L203(**-129**)	F19(**-107**)	G127(**-78**)
4	S290(**-107**)	V39(**-59**)	E3(**-40**)	R180(**-81**)	I41(**-117**)	G113(**-30**)	R206(**-96**)	E22(**-106**)	L52(**-49**)
5	R92(**-105**)	D23(**-51**)	K282(**-35**)	V116(**-78**)	D1(**-115**)	A106(**-30**)	L126(**-84**)	I32(**-88**)	Q123(**-49**)
6	A291(**-86**)	L17(**-50**)	K146(**-26**)	R119(**-77**)	E11(**-112**)	Q117(**-26**)	L52(**-65**)	A42(**-80**)	Q46(**-47**)
7	W276(**-85**)	I31(**-48**)	V283(**-19**)	E179(**-76**)	L34(**-102**)	V116(**-25**)	Q201(**-61**)	I31(**-56**)	L126(**-39**)
8	E266(**-85**)	D1(**-46**)	P10(**-14**)	A192(**-65**)	I32(**-88**)	Q246(**-12**)	G200(**-36**)	E11(**-54**)	Q4(**-28**)
9	Q284(**-81**)	Q15(**-45**)	A-4(**-7**)	L198 (**-51**)	V36(**-87**)	A237(**-10**)	R217(**-34**)	G33(**-45**)	T130(**-25**)
10	L279(**-81**)	K16(**-43**)	E11(**-6**)	P183(**-45**)	A42(**-85**)	Q249(**-7**)	A124(**-25**)	D7(**-42**)	R226(**-20**)

Values in bold and parentheses are BFE per residue in kJ/mol. Aβ and Enx suffix indicates the molecular complex
